# Post-Operative All-Cause Mortality in Elderly Patients Undergoing Abdominal Emergency Surgery: Role of Charlson Comorbidity Index

**DOI:** 10.3390/healthcare9070805

**Published:** 2021-06-26

**Authors:** Fabio Fabbian, Alfredo De Giorgi, Silvia Ferro, Domenico Lacavalla, Dario Andreotti, Simona Ascanelli, Stefano Volpato, Savino Occhionorelli

**Affiliations:** 1 Department of Medical Sciences, University of Ferrara, 44121 Ferrara, Italy; stefano.volpato@unife.it; 2 Department of Internal Medicine, St. Anna University Hospital, 44124 Ferrara, Italy; degiorgialfredo@libero.it; 3 Department of Translational Medicine, University of Ferrara, 44121 Ferrara, Italy; frrslv1@unife.it (S.F.); cchsvn@unife.it (S.O.); 4 Department of Surgery, Acute Care Surgery Service, St. Anna University Hospital, 44124 Ferrara, Italy; d.lacavalla@ospfe.it (D.L.); ndrdra@unife.it (D.A.); 5 Department of Surgery, General Surgery, St. Anna University Hospital, 44124 Ferrara, Italy; s.ascanelli@ospfe.it

**Keywords:** comorbidity, Charlson comorbidity index, all-cause mortality, emergency abdominal surgery

## Abstract

(1) Background: The Charlson comorbidity index (CCI) score has been shown to predict 10-year all-cause mortality, but its validity is a matter of debate in surgical patients. We wanted to evaluate CCI on predicting all-cause mortality in elderly patients undergoing emergency abdominal surgery (EAS); (2) Methods: This retrospective single center study included all patients aged 65 years or older consecutively admitted from January 2017 to December 2019, who underwent EAS and were discharged alive. CCI was calculated by using of the International Classification of Diseases, 9th Revision, Clinical Modification codes. Our outcome was all-cause death recorded during the 20.8 ± 8.8 month follow-up; (3) Results: We evaluated 197 patients aged 78.4 ± 7.2 years of whom 47 (23.8%) died. Mortality was higher in patients who underwent open abdominal surgery than in those treated with laparoscopic procedure (74% vs. 26%, *p* < 0.001), and in those who needed colon, small bowel, and gastric surgery. Mean CCI was 4.98 ± 2.2, and in subjects with CCI ≥ 4 survival was lower. Cox regression analysis showed that CCI (HR 1.132, 95% CI 1.009–1.270, *p* = 0.035), and open surgery (HR 10.298, 95%CI 1.409–75.285, *p* = 0.022) were associated with all-cause death independently from age and sex; (4) Conclusions: Calculation of CCI, could help surgeons in the preoperative stratification of risk of death after discharge in subjects aged ≥65 years who need EAS. CCI ≥ 4, increases the risk of all-causes mortality independently from age.

## 1. Introduction

Comorbidities are common in patients undergoing emergency general surgery [1], and they contribute to increase complexity especially in patients older than 60 years [2]. Bentrem et al. [3] analysed 24,747 operations, represented by upper gastrointestinal tract, hepatobiliary or pancreatic, and colorectal operations, demonstrating that subjects older than 75 years had higher perioperative morbidity and mortality than younger patients after adjusting for differences in preoperative comorbidities. Irrespective of procedure type, elderly patients were more likely to experience myocardial, pulmonary, and renal complications [3]. The latter data suggest that perioperative complications should include acute medical conditions in addition to surgical ones in aged. Moreover, in older adults a further severe complication should be considered after surgery, that is post-operative delirium [4]. Its consequences are longer hospitalisation, a doubling of mortality and almost all cases develop permanent, yet subtle, cognitive deficits specific to everyday life [4]. In 2018, Eamer et al. [5] reviewed risk assessment tools in studies on elderly surgical patients, and they concluded that the majority of them are not commonly used for pre-operative risk assessment. Marwell et al. [6] reported that geriatric subjects who need surgical care should undergo (i) assessments of function, mobility, cognition, and mental health; (ii) reviews of medical conditions and medications; and (iii) discussion of risks, preferences, and goals of care. The use of a risk scoring system would help physicians and surgeons in their evaluation and an index that could be objective, accurate, inexpensive, and simple to perform, based entirely on information available preoperatively, would be particularly suitable for patients who need emergency surgery. The Charlson comorbidity index (CCI) [7], a prognostic score developed and validated to predict one-year mortality in medical patients, has the advantage of including preoperative variables exclusively.

The predictive role of CCI in surgical patients is a matter of debate, with studies showing a predictive role [8,9] and others that did not confirm such a role [10], therefore we designed a retrospective single center cohort study was to evaluate the impact of CCI on predicting all-cause mortality in elderly patients who underwent emergency abdominal surgery.

## 2. Materials and Methods

### 2.1. Population and Administrative Data Source

This retrospective cohort study was conducted in agreement with the declaration of Helsinki of 1975, revised in 2013. In order to maintain data anonymity and confidentiality, patient identifiers were cancelled before data analysis, deleting the possibility of identification of subjects, either in this paper or in the database. The study was managed in agreement with the existent Italian disposition-by-law (G.U. n.76, 31 March 2008).

Data were derived from the discharge hospital records (DHR) that included gender, date of birth, date and department of hospital admission and discharge, vital status at discharge, length of stay, main and up to 6 accessory discharge diagnoses, and the most important diagnostic procedures, based on the International Classification of Diseases, 9th Revision, Clinical Modification (ICD-9-CM).

We included all patients aged 65 years or older consecutively admitted from 1 January 2017 to 31 December 2019, who underwent emergency abdominal surgery and discharged alive. Authors excluded subjects who died during admission, in-hospital death was not our main outcome, moreover such an event was cause of incompleteness of data at the time of filling DHR (*n* = 10). Authors also excluded subjects who suffered trauma, abdominal aortic aneurism repair, thoracic, orthopaedic, skin and soft tissue, urology surgery and any obstetric or gynecological procedures, and cases with incomplete data. Our outcome was all-cause death recorded during the two-year (20.8 ± 8.8 months) follow-up by institutional electronic databases.

### 2.2. Charlson Comorbidity Index (CCI) Calculation

The original CCI [7] was calculated. The index is based on the mortality rates of patients admitted to the general internal medicine service. It predicts survival in patients with multiple comorbidities, in fact each condition has different weights, and the sum of the single scores associated with the presence of a disease, is representative of a measure of total comorbidity burden. Overall, several diseases are included, i.e., myocardial infarction, congestive heart failure, peripheral vascular disease, cerebrovascular disease, dementia, chronic obstructive pulmonary disease, connective tissue disease, peptic ulcer disease, liver disease, diabetes mellitus, hemiplegia, moderate to severe chronic kidney disease, solid tumor, leukemia, lymphoma, AIDS. Additionally, prevalence of hypertension was recorded. When the score is 0, the corresponding estimated 10-year survival rate is 98%, if the final total score is 4, it suggests a 53% estimated 10-year survival, while if the total score is ≥7, the corresponding 10-year survival rate is 0% [7]. Administrative data were used in order to calculate CCI, following guidelines suggested by Quan et al. [11].

### 2.3. Statistical Analysis

We performed a descriptive analysis of all data collected, and results were expressed as absolute numbers, percentages, and mean ± SD. Univariate analysis was carried out for detecting the difference between survivors and deceased subjects; based on type of data, statistical analysis was conducted with Chi-square test for detecting difference in frequencies, and with Student *t*-test, and Mann–Whitney test for detecting differences between normally distributed data or non-normally distributed ones, respectively. Surgery was analysed as laparoscopic or open abdominal procedures and accounting for organ or apparatus involved. Therefore, we considered colon, small bowel, gallbladder, gastric surgery, hernia repair, and appendectomy. We classified as other, different types of abdominal surgery. Taking into consideration comorbidity, population was stratified in two groups, based on the median values of CCI. Survival after different types of surgery and of patients with CCI< or ≥median value was estimated by Kaplan–Meier curves. Cox regression analysis for hazard ratios (HR) calculation was performed, age, sex, and CCI were the independent variables. The survival analysis was performed using the Kaplan–Meier method with the log-rank test. All *p*-values were two-tailed, with significance defined as *p* < 0.05. Statistical analysis was performed using IBM SPSS Statistics version 26 (IBM Corp., Armonk, NY, USA).

## 3. Results

**Figure 1:**
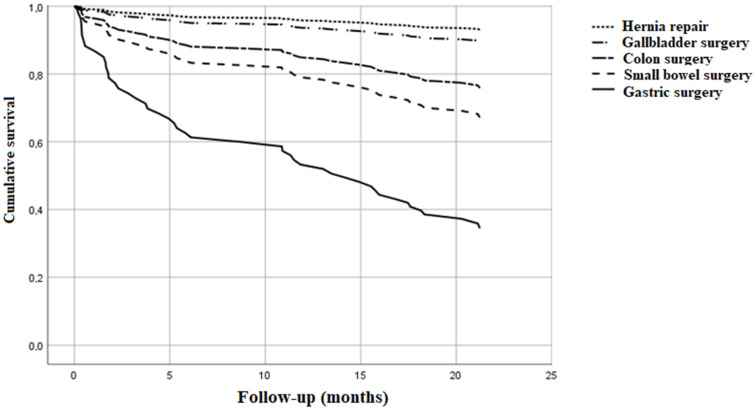
Cumulative survival of individuals undergoing emergency abdominal surgery by the type of surgery. None of the subjects undergoing appendectomy died during follow-up.

During the study period of 20.8 ± 8.8 months, we enrolled 197 Caucasian patients aged 78.4 ± 7.2 years (102 women). Survivors represented 150 (76.2%) participants and deceased represented 47 (23.8%) participants; fortunately we did not miss any case during the follow-up of 20.8 ± 8.8 months. Laparoscopic procedures were carried out in 40 subjects (20.3%) and open abdominal procedures in 157 (79.7%), and their comorbidity burden was different being higher in those who underwent open surgery (CCI 5.27 ± 2.28 vs. 3.85 ± 1.36, *p* < 0.001). The cohort underwent the following operations: colon surgery in 46 patients (23.4%), small bowel surgery in 53 (26.9%), gallbladder surgery in 62 (31.5%), hernia repair in 19 (9.6%), appendectomy in 9 (4.6%), gastric surgery in 6 (3%), and other in 2 (1%). The six gastric surgeries were all open and included 4 gastrectomy and gastric resections and 2 ulcer repair interventions. Mortality was higher in patients who underwent open abdominal surgery than in those treated with laparoscopic procedure (74% vs. 26%, *p* < 0.001), and in those who needed colon, small bowel and gastric surgery (Figure 1). Mean CCI was 4.98 ± 2.2, median value 4 and range 2 to 15. CCI was lower than 4 in 54 patients and ≥4 in 143 (71.9%). Table 1 shows clinical features of survivors and deceased subjects based on ICD-9-CM diagnostic codes. None of the patients had AIDS. Prevalence of hypertension, heart, pulmonary, peripheral vascular, connective tissue, peptic ulcer, liver, and chronic kidney disease, diabetes, blood neoplasms, and solid tumor without metastasis were not different in the two groups. Moreover, dementia was not different in survivors and deceased groups, although its prevalence was unexpectedly low. On the contrary non-survivors were older, had longer length of hospital stay, higher prevalence of hemiplegia, solid tumor with metastasis, and higher comorbidity score than people who were alive at the end of follow-up.

**Figure 2:**
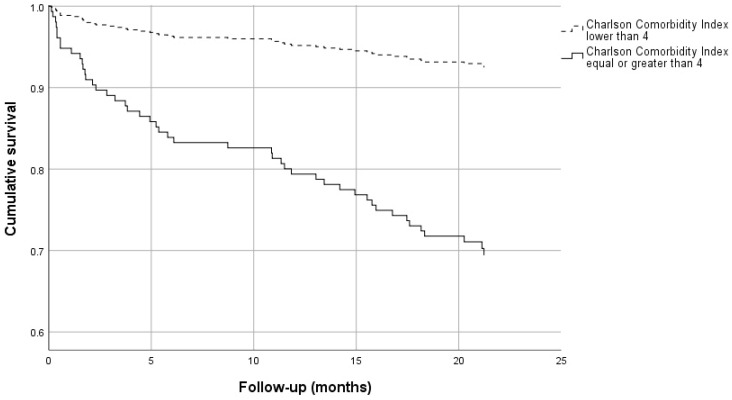
Cumulative survival of individuals undergoing emergency abdominal surgery with Charlson comorbidity index <4 and ≥4.

Finally, cerebrovascular disease was marginally higher in deceased group. Cox regression analysis showed that CCI (Hazard Ratio 1.132, 95% Confidence interval 1.009–1.270, *p* = 0.035), and open surgery (Hazard Ratio 10.298, 95% Confidence interval 1.409–75.285, *p* = 0.022) were independently associated with all-cause death while age and sex were not (Table 2). Survival of patients with CCI lower than 4 and ≥4 is shown in Figure 2.

## 4. Discussion

In this study involving elderly patients, CCI is associated with all-cause mortality after discharge post-emergency abdominal surgery, independent of age and gender. A simple score such as CCI, ref. [7] exclusively based on preoperative variables, is associated with a negative outcome, confirming previous findings [8]. Mehta et al. [12] reported that comorbidity scores could predict surgical outcomes such as 30-day mortality, 1-year mortality, 30-day readmission, complications and failure to rescue, with a c-statistic > 0.7 [12]. We found that for every increasing unit in CCI, the risk of all-cause mortality after discharge rises of 16.9% during a mean period of about 21 months, and that a CCI equal or greater than 4 is a major risk factor for all-cause death. Moreover, the predictive value was independent from the age of patients. This latter finding suggests that age per se is not a predictor of negative outcome after emergency abdominal surgery.

The influence of comorbidities on the survival of surgical patients could involve several mechanisms. Comorbidities increase the risk of death during the follow-up for reasons other than surgical intervention per se, although it is possible that surgical treatment may alter the balance between different conditions involving different organs. On the other hand, the mechanism due to surgical procedure that could directly influence the different comorbidities is still unclear. Comorbidity is a determinant of the type of surgical procedure (i.e., laparoscopic or open) and of long term outcome in patients aged 65 years or older undergoing emergency abdominal surgery. Our data show that calculation of a simple comorbidity index, such as CCI, could help surgeons in prognosis evaluation.

A public health report of United States published in 2014, showed that more than 7% of all hospitalisations were due to urgent general surgery admissions, and comorbidity was a frequent finding; indeed, more than 50% of the study population (about 27 million of emergency general surgery) had at least one chronic comorbid condition [2]. In older adults who need emergency general surgical treatment, more than 50% have three or more chronic diseases and poor general conditions are associated with many adverse consequences. Therefore, focusing on the management of a single disease in adults with multimorbidity could fail if single conditions clinical practice guidelines are applied [13]. Barazanchi et al. [14] conducted a scoping systematic review aiming at examining pre-operative risk factors for mortality in subjects who underwent emergency laparotomy. Their outcome was post-operative 30-day mortality that was 13% out of more than 157,000 patients included in the 22 studies analysed. Risk factors included age, American Society of Anestesiologists score, preoperative sepsis, patient functional-dependency status, do-not-resuscitate patient choice, and preoperative ventilator dependence. Mortality was not affected by in-hospital delays to surgery, diabetes mellitus, gender, and specialist role of the surgeon performing the emergency surgery [14]. Medical comorbidity was marginally considered. We evaluated the relationship between medical conditions at the time of emergency surgery and all-cause death during a mean 21-month follow-up. In 2015, Chiulli et al. [15] conducted a retrospective review analysing patients from general vascular surgery and subspecialty in a tertiary referral center. Authors found that failure of surgery was associated with preoperative congestive heart failure, renal failure, and ascites, suggesting the importance of CCI calculation, indeed, in this index, congestive heart failure scores 1, kidney failure scores 2, and liver failure scores 3. However, Ho et al. [16] reported different finding, they evaluated mortality in a large population of patients aged 65 years or older operated emergently. They showed that coagulopathy, fluid, and electrolyte disorders, and liver disease were associated with the highest mortality. In a recent study from Italy, high pre-operative CCI measure was associated with major post-operative complications and mortality in 259 consecutive subjects aged 80 ± 8 years who underwent emergency surgery for complicated inguinal or femoral hernia [8]. Laor et al. [9] compared the impact of age and CCI on 30-day and one-year mortality in patients aged 75–84 years and subjects aged ≥85 years. Increasing age and CCI were associated with death after elective and emergency surgery; however, CCI was superior to age as a predictor of early and late mortality. Massarweh et al. [17] demonstrated the impact of age on post-surgical complications risk. The 90-day cumulative incidence of complications in adults aged more than 65 years who underwent common abdominal procedures, such as cholecystectomy, colectomy, and hysterectomy, was 17.3%, with a 90-day mortality rate of 5.4%. Frequency of complications increased from 14.6% in patients aged 65–69 years to 22.7% in those aged ≥90 years. Shah et al. [18] evaluated 6,712,151 discharge records for emergency general surgery with mean age of 58.5 years. Uninsured patients were more likely to die and advanced age was an independent predictor of mortality. Patients requiring resuscitation, and those with vascular and hepatic disease had the highest likelihood of mortality. In our study, although deceased group had higher age, the latter was not associated with all-cause mortality, suggesting a different impact of age and comorbidity in causing negative outcomes. Similarly to our study, Arenal et al. [19] found that in a population of more than 700 subjects aged ≥70 years who suffered an abdominal emergency surgery operation, increasing age did not affect mortality, morbidity or length of hospital stay. On the other hand, CCI was not a predictor of operative morbidity and mortality in non-agenarian patients, however, authors calculated that cut-off values of 3.5 and 4.5 had a relationship with operative mortality and post-operative complications, respectively [10]. These cut-off values are similar to the median CCI calculated in our study and able to differentiate non-survivors from survivors during follow-up.

### Limitations

We need to mention some limitations. First, this is a single center study, evaluating exclusively patients admitted to emergency surgery ward, and conducted on a small population. Moreover, our population was not homogeneous, in fact we analyzed patients with higher comorbidity burden who underwent open surgery and subjects with lower comorbidity burden who underwent laparoscopic surgery. Therefore, results may not be generalisable. Second, low sensitivity and specificity are the major limitations of studies based on ICD-9-CM codes. The latter are mainly related to the financial purposes. Third, we evaluated only all-cause mortality, and we did not analyze short-term and long-term operative complications, but we only considered all-cause mortality. On the other hand, long term all-cause mortality is a strong outcome aiming at the evaluation of effectiveness of interventions. Fourth, our study did not include clinical features, we based our conclusions merely on the burden of comorbidity, calculated by ICD-9-CM codes. The latter do not include clinical severity, functional and cognitive status, or intensity of care given. In the same way some comorbidities could not had been accounted for, because of lack of recording, such as in the case of dementia. Accuracy and completeness of data are essential for calculating the true comorbidities index, therefore low quality data could reduce the performance of a comorbidity score and its ability to predict the outcome [20]. Finally, we did not evaluate frailty.

## 5. Conclusions

The proportion of elderly subjects needing surgery is increasing and they have higher burden of comorbidity compared with their younger counterparts, they have longer hospital stay, higher frequency of complications and intensive care unit admissions, and higher mortality both peri-operation and post-operation. In elderly patients, it is difficult to consider every single risk factor for negative outcome, especially in an emergency surgical care setting; for this reason, a general assessment including medical chronic conditions could be useful in the management of surgical patients [21]. In elderly subjects, comorbidity is a risk factor for in-hospital mortality [22] and CCI has been associated with development of nosocomial infections [23] and it has been used for evaluation preoperative clinical status of older adults who need laser lithotripsy [24]. Comorbidity scores are usually easy to calculate and summarise in a unique number the burden of comorbidities predicting short and long-term prognosis, independently from age. We think that calculation of a medical comorbidity score, such as CCI, could help surgeons in the pre-operative stratification of risk of death after discharge in subjects aged ≥65 years who need emergency abdominal surgery. High burden of comorbidity, i.e., CCI ≥ 4, increases the risk of all-causes mortality independently from age.

## Figures and Tables

**Table 1:** Clinical features of patients undergoing emergency abdominal surgery that survived and deceased.

	Survivors	Deceased	*p*
	(*n* = 150)	(*n* = 47)	
Age (years)	77.4 ± 6.4	81.5 ± 7.7	<0.01 *
Women (*n* (%))	76 (50.7)	25 (53.2)	0.867 °
Men (*n* (%))	74 (49.3)	22(46.8)	
Length of hospital stay (days)	10.6 ± 6.5	14.2 ± 9.2	<0.01 ^
Hypertension (*n* (%))	106 (70.6)	34 (72.3)	1.000 °
Myocardial infarction (*n* (%))	24 (16)	5 (10.6)	0.481 °
Congestive heart failure (*n* (%))	19 (12.7)	5 (10.6)	0.804 °
Peripheral vascular disease (*n* (%))	25 (16.7)	7 (14.9)	1.000 °
Cerebrovascular disease (*n* (%))	22 (14.7)	13 (27.6)	0.05 °
Dementia (*n* (%))	3 (2)	2 (4.2)	0.335 °
Chronic obstruction pulmonary disease (*n* (%))	17 (11.3)	4 (8.5)	0.788 °
Connective tissue disease (*n* (%))	9 (6)	3 (6.3)	1.000 °
Peptic ulcer disease (*n* (%))	5 (3.3)	1 (2.1)	1.000 °
Diabetes mellitus (*n* (%))	16 (10.7)	6 (12.8)	0.582 °
Chronic kidney disease (*n* (%))	0 (0)	2 (4.3)	0.056 °
Hemiplegia (*n* (%))	1 (0.6)	3 (6.3)	0.041 °
Leukemia and/or malignant lymphoma (*n* (%))	4 (2.7)	3 (6.4)	0.361 °
Solid tumor without metastasis (*n* (%))	10 (6.7)	5 (10.6)	0.358 °
Solid tumor with metastasis (*n* (%))	3 (2)	8 (17)	0.001 °
Liver disease (*n* (%))	8 (5.3)	2 (4.2)	1.000 °
AIDS (*n* (%))	0 (0)	0 (0)	-
Charlson comorbidity index	4.5 ± 1.9	6.3 ± 2.4	<0.001 ^
Charlson comorbidity index ≥ 4 (*n* (%))	100 (66.7)	43 (91.5)	0.001 °

* Student t-test; ° Chi-square test; ^ Mann–Whitney test.

**Table 2:** Cox regression analysis showing that Charlson comorbidity index (CCI) and open surgery were independently associated with mortality during follow-up.

	Hazard Ratio	Lower 95% Confidence Interval	Higher 95% Confidence Interval	*p*
Age	1.039	0.990	1.091	0.120
Sex	0.960	0.540	1.708	0.890
CCI	1.132	1.009	1.270	0.035
Open surgery	10.298	1.409	75.285	0.022

## Data Availability

The data presented in this study are available on request from the corresponding authors.
